# Longitudinal Invariance Analysis of the Short Grit Scale in Chinese Young Adults

**DOI:** 10.3389/fpsyg.2020.00466

**Published:** 2020-03-24

**Authors:** Jie Luo, Meng-Cheng Wang, Ying Ge, Wei Chen, Shuang Xu

**Affiliations:** ^1^School of Psychology, Guizhou Normal University, Guiyang, China; ^2^Department of Psychology, Guangzhou University, Guangzhou, China; ^3^The Key Laboratory for Juveniles Mental Health and Educational Neuroscience in Guangdong Province, Guangzhou University, Guangzhou, China; ^4^The Center for Psychometrics and Latent Variable Modeling, Guangzhou University, Guangzhou, China; ^5^Key Laboratory of Emotion and Mental Health in Chongqing, Chongqing Collaborative Innovation Center for Brain Science, Chongqing University of Arts and Sciences, Chongqing, China

**Keywords:** longitudinal measurement invariance, personality trait, grit, Grit-S, Chinese young adults

## Abstract

The current study examined the longitudinal measurement invariance (LMI) of the Short Grit Scale (Grit-S) in a survey sample of Chinese young adults (*N* = 233, 48.9% male, mean age = 19.36 years, *SD* = 0.90 years) who completed the Grit-S twice over a 3-month interval. Confirmatory factor analysis was conducted to examine the LMI of the Grit-S across time. Results showed that the Grit-S has strict longitudinal invariance (i.e., equality of factor patterns, factor loadings, item intercepts, and item uniqueness for all items) over time. Additionally, the internal consistency indices of the Grit-S were acceptable across time, the stability coefficients over time were moderate, and latent factor means did not differ significantly across time. In sum, these findings suggest that the Grit-S has satisfactory longitudinal properties when used in Chinese young adults.

## Introduction

Grit, as a personality trait, is interpreted as trait-level perseverance with a passion for long-term goals, and it has been shown to predict an individual’s achievement in challenging domains over and beyond measures of talent (Duckworth et al., 2007; Duckworth and Quinn, 2009). According to Duckworth et al. (2007), although there has been some empirical evidence for a close relationship between grit and conscientiousness (e.g., Ivcevic and Brackett, 2014; Rimfeld et al., 2016; Schmidt et al., 2018), grit distinguishes from the traditionally measured facets of Big Five Conscientiousness in its focusing on stamina. More specifically, grit shows that one is available to keep effort and interest in projects that may take months or even more to accomplish. Individuals with high scores in grit measurement do not stray from their goals, even without positive feedback (Duckworth and Quinn, 2009). Moreover, grit is also related to or overlaps with self-control (Credé et al., 2017; Vazsonyi et al., 2018; Werner et al., 2019), yet it differs from self-control or self-regulation (Duckworth and Quinn, 2009; Duckworth and Gross, 2014). According to Duckworth and Gross (2014), self-control entails the ability to sustain focus on a present task and to desist from distractions, more consistent with avoidance systems; grit, on the other hand, is best understood as an ability to pursue long-term goals and is related to the approach motivation system. As such, grit is unique and should remain predictive independent of self-control because it focuses on the ability to attain long-term goals (Duckworth and Gross, 2014).

Traditionally, grit researchers conceptualized grit as the combination of two components: perseverance of effort (PE) and consistency of interests (CI). Despite the extensive studies of grit as a whole construct and obtaining a total scale score by summing the PE and CI subscale scores, there is an increasing amount of evidence that the two grit facets can reflect independent constructs instead of aspects of the single grit construct (e.g., Credé et al., 2017; Tyumeneva et al., 2017). For instance, prior research has shown the unique validity of two grit subscales for performance outcome (Credé et al., 2017) as well as well-being and personality strength (Disabato et al., 2018). Accordingly, researchers recommended that the two grit subscales (i.e., PE and CI) be kept separate instead of combining them to form a total grit score (Credé, 2018). Broadly speaking, growing evidence has shown that grit may predict one’s success and performance in academic, vocational, and avocational domains (Duckworth et al., 2007, 2009; Duckworth and Quinn, 2009; Eskreis-Winkler et al., 2014; Zhong et al., 2018). For example, at the United States Military Academy in West Point, New York, freshman cadets who measured higher in grit were less likely to drop out than their less-gritty peers, even after controlling for other measures (e.g., SAT scores, high school rank, and Big Five Conscientiousness) (Duckworth et al., 2007). Additionally, a recent meta-analysis has indicated that overall grit exhibited a relationship with overall academic performance as well as with overall GPA criterion (Credé et al., 2017). Likewise, existing meta-analytic evidence indicates differences between the two grit facets in predicting achievement, retention, and intelligence outcomes (Credé et al., 2017). In summary, given the importance of grit in educational, personal, and professional domains, it is therefore necessary to identify and validate the brief, stand-alone measure of grit.

### The Grit Scale and Its Short Version

In the absence of adequate existing measures, Duckworth et al. (2007) developed and validated the 12-item self-report measure of grit (Grit-O). The Grit-O was theoretically consistent with grit as a compound trait comprising stamina in the dimensions of interest (i.e., consistency of interest) and effort (i.e., PE). The consistency of interest (CI) factor refers to the tendency to not change goals and interests frequently; the PE factor assesses the tendency to work hard even in the face of setbacks. Duckworth et al. (2009) subsequently revised and developed a more economical and efficient measure of grit: the Short Grit Scale (Grit-S). The Grit-S keeps the proposed two-factor structure of the full Grit-O, but contains four fewer items and demonstrates better psychometric properties than the original Grit-O (Duckworth and Quinn, 2009).

Following the work of Duckworth et al. (2009), the Grit-S has been formally translated into Japanese (Nishikawa et al., 2015), Turkish (Sarıçam et al., 2015), Filipino (Datu et al., 2016), German (Schmidt et al., 2017), Polish (Wyszyńska et al., 2017), Spanish (Arco-Tirado et al., 2018), and Chinese (Wang et al., 2017; Li et al., 2018; Zhong et al., 2018). Overall, cross-sectional data show that each version of the Grit-S has acceptable psychometric properties and that each translation resembles the English-speaking version (see Table 1). None of these investigations, however, have assessed the longitudinal properties for the Grit-S, nor have they focused on the longitudinal measurement invariance (LMI) of the Grit-S scores over different time periods.

**TABLE 1:** Psychometric properties in previous studies for the Grit-S.

Authors	Sample characteristics	Country	Method	Best model	α (number of items)	Fit indices
Nishikawa et al., 2015	994 university students: 52.1% female, *M* = 18.93, *SD* = 0.99	Japan	EFA	Two-factor model	PE 0.78(4), CI 0.73(4)	
Sarıçam et al., 2015	186 university students: 58.1% female, *M* = 21.3	Turkey	CFA	Two-factor model	Total 0.83(8), CI 0.80(4), PE 0.71(4)	CFI = 0.95, RMSEA = 0.046
Datu et al., 2016	***Sample One***220 college students: 67.7% female, *M* = 18.22, *SD* = 1.58 ***Sample Two***606 high school students: 49.5% female, *M* = 13.87, *SD* = 1.26	Philippines	CFA	Two-factor model	***Sample One***CI 0.61(4), PE 0.58(4) ***Sample Two***CI 0.63(4), PE 0.60(4)	***Sample One***CFI = 0.97, RMSEA = 0.05***Sample Two***CFI = 0.94, RMSEA = 0.06
Schmidt et al., 2017	525 university students: 72.1% female, *M* = 27.93, *SD* = 3.63	Germany	CFA	Modified high-order model	Total 0.80(8)	CFI = 0.99, TLI = 0.99, RMSEA = 0.03
Wang et al., 2017	217 high school graduates: 53.0% female, *M* = 18.48, *SD* = 0.55	China	CFA	Two-factor model	Total 0.81(8)	CFI = 0.98, TLI = 0.97, RMSEA = 0.046
Wyszyńska et al., 2017	270 adults: aged 18–34 years, 52.4% female, *M* = 20.79	Poland	CFA	Two-factor model	CI 0.72(4), PE 0.69(4)	CFI = 0.979, RMSEA = 0.038
Arco-Tirado et al., 2018	1,826 adults: aged 18–35 years, 51.1% female, *M* = 27.56, *SD* = 5.00	Spain	CFA	One-factor model	Total 0.75(8), CI 0.77(4), PE 0.48(4)	CFI = 0.95, RMSEA = 0.071
Li et al., 2018	607 adolescents: 58.3% female, *M* = 17.1, *SD* = 0.50	China	CFA	Two-factor model	Total 0.80(8), CI 0.78(4), PE 0.72(4)	CFI = 0.98, RMSEA = 0.05
Zhong et al., 2018	2,363 adults: aged 19–70 years, 62.7% female, *M* = 35.14, *SD* = 8.99	China	EFA, CFA	Two-factor model	Total 0.85(8), CI 0.70(4), PE 0.75(4)	CFI = 0.986, TLI = 0.979, RMSEA = 0.06

*EFA, exploratory factor analysis; CFA, confirmatory factor analysis; CFI, comparative fit index; TLI, Tucker-Lewis index; RMSEA, root mean square error of approximation; CI, consistency of interest; PE, perseverance of effort.*

While the Grit-S is a popular measurement for grit, there have been some controversies regarding the factor structure of the Grit-S. More specifically, the original factor structure of the Grit-S was a high-order construct with two low-order components (i.e., PE and CI) and was based on confirmatory factor analysis (Duckworth and Quinn, 2009). Some comments, however, have suggested that this solution might be problematic (e.g., Credé et al., 2017; Credé, 2018) since a factor model with one second-order factor and two first-order factors cannot be identified at the higher-order level (Kline, 2011). Criterion-related studies, on the other hand, with Grit-S also have inconsistencies – they either combine the two grit facets to a single grit score (Duckworth and Quinn, 2009) or treat the two grit subscales separately (Credé, 2018; Guo et al., 2019). Additionally, recent controversies have focused on how the Grit-S captures only perseverance (PE) without passion (CI) (e.g., Jachimowicz et al., 2018, 2019; Credé, 2019; Guo et al., 2019). Given that prior studies that examined the psychometric properties of the Grit-S preferred the two first-order factors structure to the high-order factor solution (see Table 1), the present study would like to examine the longitudinal properties of the Grit-S within the two lower order factors model.

### Measurement Invariance of the Grit-S

Measurement invariance (MI) is vital because the interpretation of mean differences may be misguided and questionable unless there is the same latent construct in different subgroups (Byrne and Watkins, 2003; Chen, 2008). That is, the establishment of MI is a prerequisite for meaningful comparisons across groups (e.g., male vs. female) (Chen, 2008). Previous studies (Datu et al., 2016; Schmidt et al., 2017; Zhong et al., 2018) have discussed the MI of the Grit-S scores for gender, educational levels, and age groups. For example, Zhong et al. (2018) showed that the self-report measure of Grit-S has strict MI across gender and age in Chinese insurance employees. Likewise, the partial strict invariance across gender and different levels has been supported using a German sample of university students (Schmidt et al., 2017). In a mixed sample of Filipino high school and university students, only the configural invariance model was supported, while not existing evidence of measurement and structural invariance when comparing between two student groups (i.e., high school and university students; Datu et al., 2016).

While existing research has focused on the MI of the Grit-S across different groups (e.g., gender and age), the LMI (i.e., measurement invariance across different points in time) for Grit-S has not been explored. Similar to the MI across different groups, LMI tests the equality of a construct for an instrument, but its focus is on equality across time rather than across groups (Dimitrov, 2010; Millsap and Cham, 2012). LMI is a desirable quality in a measurement because it indicates that the same construct can be tested across occasions (i.e., configural, metric, scalar, and strict invariance), providing a solid and necessary basis for mean comparisons in longitudinal studies. Any inference about developmental changes over time may be misleading and inaccurate unless the premise of LMI is met (Dimitrov, 2010; Millsap and Cham, 2012). As such, confirming the LMI is critical to be able to draw valid conclusions about growth and changes in latent constructs across time. Although longitudinal studies that examine the relationship between grit and other covariates across diverse situations have been common in health and occupational psychology (e.g., Duckworth et al., 2007, 2009, 2011; Duckworth and Quinn, 2009), these studies did not measure whether grit has MI across time. In the current research, it is the first time to test whether the Grit-S has LMI over time.

### The Present Study

The main purpose of this research was to examine the LMI of Grit-S in a survey sample of Chinese young adults. For this purpose, the confirmatory factor analysis was conducted to test whether the Grit-S scores have LMI. Specifically, we tested the configural, metric, scalar, and strict invariance over a 3-month interval. Given that traits such as grit describe tendencies to act, think, and feel that are relatively stable across time and situations (Duckworth et al., 2007), it could be expected that the Grit-S scores would have strict longitudinal invariance. The internal consistency values of the Grit-S scores were measured separately, first at the baseline and then at the follow-up. Finally, the stability coefficients across time were computed, and the latent factor means from both times were compared.

## Materials and Methods

### Participants

The subjects used in the current investigation were recruited from a normal university in Guiyang city, China. In this in-progress longitudinal research, we aimed to seek a more particular knowledge of the correlates and causes of heterogeneity in freshman adaptation to college and psychological health. The first survey was administered at the beginning of the second semester of freshman year in March 2019, when 296 first-year students were recruited to complete the Chinese version of the Grit-S (Zhong et al., 2018); the second assessment was conducted in the end of the second semester of the freshman year (June 2019), with 233 of the original first-year students attending the investigation. Participant data from those subjects who did not complete the second survey were excluded (*n* = 63). An independent-samples *t*-test showed that the two subscales and total scale scores of the Grit-S at Time 1 were not significantly different between the participants and dropouts at Time 2 (CI: *t* = −0.147, *p* = 0.883; PE: *t* = 0.133, *p* = 0.894; Grit-S total: *t* = −0.005, *p* = 0.996), suggesting that the sample attrition at Time 2 was random. Regarding the final sample, participants were between 17 and 22 years of age (*M* = 19.36, *SD* = 0.90), with approximately 91% of participants being 18–20 years old, 114 (48.9%) were male and 119 (51.1%) were female, 83 (35.6%) were majoring in education, 38 (16.3%) were majoring in science, 40 (17.2%) were majoring in economics, 15 (6.4%) were majoring in law, and 57 (24.5%) were majoring in engineering. Most participants were of Han ethnicity (79%), with the remaining 21% being of mixed ethnic minority backgrounds. Finally, a statistical power analysis indicated that a sample size of 190 would be needed for power of 0.80 by a Monte Carlo study in a confirmatory factor analysis model (Muthén and Muthén, 2002). Moreover, G^∗^power 3.1.9.2 (Faul et al., 2009) suggested that a sample size of 35 would be needed to obtain a satisfactory test-retest coefficient (*r* = 0.70, α = 0.01, 1-β = 0.99) within an interval of time, and a sample size of 100 would be needed to detect a medium effect size (*d* = 0.5, α = 0.01, 1-β = 0.99) between two times. A final sample size (*n* = 233) would be used to test the longitudinal properties of the Grit-S over a 3-month interval between the two assessments.

### Procedure

The study questionnaires were administered in a classroom setting when participants were attending their classes. All participants provided written consent prior to completing the questionnaire, having been notified of the nature, goal, confidentiality, and anonymity of the study. The present study was approved by the Human Subjects Review Committee at Guizhou Normal University. All participants completed study questionnaires for extra course credit.

### Measures

#### The Short Grit Scale (Grit-S)

The Grit-S (Duckworth and Quinn, 2009) is a brief version of the full Grit-O (Duckworth et al., 2007) developed to measure trait-level perseverance and passion for long-term goals using two factors: consistency of interest (CI; 4-item) and perseverance of effort (PE; 4-item). Each item of the self-reported Grit-S scale is rated on a five-point Likert scale that ranges from 1 (“not at all like me”) to 5 (“very much like me”). The Chinese version of the Grit-S has been validated in adolescents (Wang et al., 2017; Li et al., 2018) and in adults (Zhong et al., 2018). In this present study, the alphas and mean inter-item correlation (MIC) for CI and PE at the two time points were 0.75 (MIC = 0.42)/0.75 (MIC = 0.43) and 0.80 (MIC = 0.49)/0.78 (MIC = 0.48), respectively.

#### Data Analysis Strategy

Firstly, descriptive statistics of the Grit-S scores were performed with SPSS 22.0 (IBM Corp, 2013). Next, following the previous longitudinal studies (e.g., Wang et al., 2012; Luo et al., 2019), the CFA with M*plus* 7 (Muthén and Muthén, 1998–2015) was used to test LMI across time. The proposed two first-order factors structure was seen as a baseline model. In this model, the eight items of the Grit-S assessed separately at Time 1 and Time 2 are loaded on the two factors (i.e., CI: 4 items, and PE: 4 items). Given that the values of the skewness and kurtosis for some items were not the range of −1 to +1, we used a maximum likelihood estimation with a mean-adjusted chi-square (MLM) that was robust to non-normality. A model is judged to have an adequate model fit if the comparative fit index (CFI) and the Tucker–Lewis index (TLI) are each larger than 0.90, and if the root mean square error of approximation (RMSEA) is smaller than 0.08; if CFI and TLI are above 0.95 and RMSEA values are below 0.05, this indicates a good model fit (Hu and Bentler, 1999).

Then, the LMI was tested across time using a set of four nested models by continuously setting the equality of the parameters of the measurement model over time. The configural invariance tests the hypothesis that the same general pattern of factor loadings holds across time (Millsap and Cham, 2012); the metric invariance sets the corresponding factor loadings to be equal across occasions; the scalar invariance requires the corresponding factor loadings and intercepts across time to be set as equal; and the strict invariance sets the corresponding factor loadings, intercepts, and residual variances of items to be equal over time. To evaluate the invariance at each level, a chi-square difference test was computed but not used due to the fact that the chi-square difference test is sensitive to minor parameter changes in large samples (Chen, 2007). Instead, the change in CFI (△CFI) was used, with changes smaller than 0.01 indicating that the more restrictive model and the less restricted model were equivalent (Cheung and Rensvold, 2002; Chen, 2007). Additionally, as recommended by Chen (2007), a change in RMSEA (△RMSEA) of 0.015 or higher suggests an absence of MI.

Next, the reliability assessment of the Grit-S was performed, including measuring the internal consistency and stability coefficient. The Grit-S internal consistency was examined by looking at the two time points individually. According to Barker et al. (1994), alpha coefficients below 0.60 suggest insufficient, 0.60–0.69 indicate marginal, 0.70–0.79 suggest acceptable, 0.80–0.89 indicate good, and above 0.90 indicate excellent. We also inspected the MIC, which are independent of scale lengths and should be in the range of 0.15–0.50 to be considered acceptable (Clark and Watson, 1995). The stability coefficients (correlations between two-time point factors) across time were also calculated by using the strict invariance model to assess the relative stability of the grit trait. Specifically, setting the factor variances to 1 and freely estimating the first factor loading for each factor made the purpose of calculating latent factor correlations.

Finally, on the basis of the LMI, the latent factor means across time were compared to explore the development of the grit trait. More specifically, the latent factor scores were calculated by setting the two grit factors mean to zero at Time 1 and freely estimating the latent factor mean at Time 2.

## Results

### Descriptive Statistics

Descriptive statistics results for each item at both time points are shown in Table 2, involving the mean, standard deviation, skewness, kurtosis, and corrected item-total correlations (CITC) with each item’s respective factor, as well as the two Grit-S subscales and the total scale. Moreover, the zero-order (observed) correlations between the subscales for the two assessments were 0.22 (Time 1) and 0.10 (Time 2), respectively.

**TABLE 2:** Descriptive statistics for the Short Grit Scale at two time points.

Item	Time 1					Time 2				
	*M*	*SD*	*SK*	*KU*	*CITC*	*M*	*SD*	*SK*	*KU*	*CITC*
Consistency of interest	3.11	0.69	−0.03	0.44		3.07	0.67	−0.11	0.70	
1. New ideas and projects sometimes distract me from previous ones	3.26	0.82	0.14	0.39	0.37	3.14	0.76	0.12	1.07	0.47
3. I have been obsessed with a certain idea or project for a short time but later lost interest.	3.04	0.98	−0.02	−0.01	0.51	3.00	0.87	−0.17	0.13	0.52
5. I often set a goal but later choose to pursue a different one.	3.10	0.95	−0.04	−0.07	0.62	3.04	0.89	−0.23	0.35	0.59
6. I have difficulty maintaining my focus on projects that take more than a few months to complete.	3.04	0.97	0.00	−0.03	0.61	3.08	0.99	−0.11	0.16	0.61
Perseverance of Effect	3.29	0.72	0.11	0.14		3.28	0.70	0.11	0.03	
2. Setbacks don’t discourage me.	3.14	0.99	−0.09	−0.33	0.48	3.22	0.88	0.02	−0.40	0.51
4. I am a hard worker.	3.36	0.93	−0.10	−0.19	0.63	3.31	0.90	0.02	−0.10	0.61
7. I finish whatever I begin.	3.26	0.87	−0.03	0.08	0.58	3.17	0.92	0.22	−0.33	0.57
8. I am diligent.	3.42	0.91	−0.06	0.02	0.70	3.42	0.89	0.03	−0.24	0.68
Total Grit-S scores	3.20	0.55	0.44	0.84		3.17	0.51	0.40	1.77	

*CITC, corrected item-total correlations with each item’s respective factor; Grit-S, the Short Grit Scale.*

### Longitudinal Measurement Invariance of the Grit-S

**FIGURE 1:**
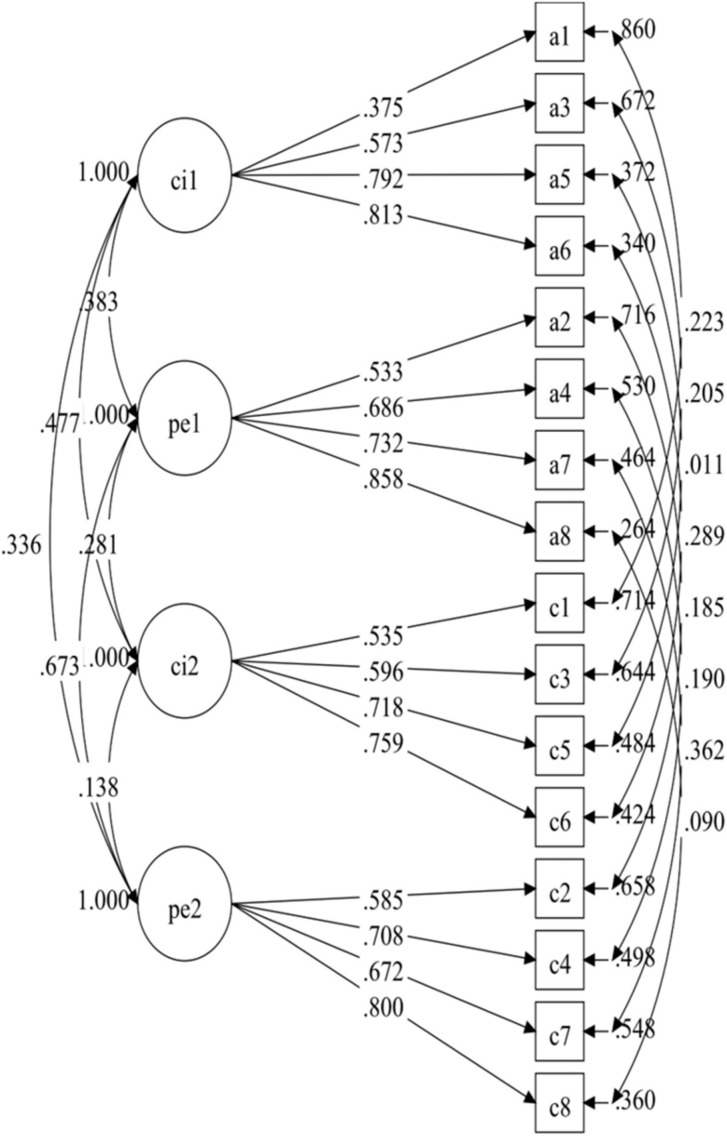
Diagram for the longitudinal configural invariance model. CI-1, consistency of interest at Time 1; PE-1, perseverance of effort at Time 1; CI-2, consistency of interest at Time 2; PE-2, perseverance of effort at Time 2.

The LMI of the Grit-S across time was calculated using the following steps. First of all, we assessed the fit of the model for each time point separately. All model fit values were adequate for both time points (CFI and TLI > 0.90, RMSEA < 0.08), allowing for further examination of the LMI. As shown in Table 3, the configural model was adequate (CFI = 0.947, TLI = 0.930, and RMSEA = 0.050). The correlations within and between factors for the model are presented in Figure 1.

**TABLE 3:** Longitudinal measurement invariance model fit statistics for the Short Grit Scale.

Model	χ2	*df*	CFI	TLI	SRMR	RMSEA (90% CI)	△χ2 (*p*)	△CFI	△TLI	△RMSEA
Time 1	72.1202	19	0.934	0.903	0.055	0.079 (0.055, 0.105)				
Time 2	35.654	19	0.972	0.959	0.041	0.049 (0.000, 0.082)				
Configural	176.996	90	0.947	0.930	0.055	0.050 (0.034, 0.065)				
Metric	182.427	96	0.949	0.936	0.059	0.048 (0.032, 0.063)	5.430 (0.4899)	0.002	0.006	−0.002
Scalar	196.590	102	0.941	0.931	0.060	0.050 (0.035, 0.064)	14.163 (0.0279)	−0.008	−0.005	0.002
Strict	211.090	110	0.938	0.933	0.063	0.049 (0.034, 0.063)	14.500 (0.0696)	−0.003	0.002	−0.001

*df, degrees of freedom; CFI, comparative fit index; TLI, Tucker–Lewis index; SRMR, standardized root mean square residual; RMSEA, root mean square error of approximation; 90% CI, 90% confidence interval around RMSEA; △χ2, change in *χ*2 relative to the preceding model; (*p*), *p* value of Δχ2; ΔCFI, change in comparative fit index relative to the preceding model; ΔTLI, change in Tucker-Lewis index relative to the preceding model; ΔRMSEA, change in root mean square error of approximation relative to the preceding model.*

Then, the factor loadings were set to be equal across time to test for metric invariance. The metric model fit was satisfactory (CFI = 0.949, TLI = 0.936, and RMSEA = 0.048), and there were inappreciable differences in CFI, TLI, and RMSEA between the configural and metric models (△CFI = 0.002, △TLI = 0.006, and △RMSEA = −0.002). These findings supported the metric invariance of the Grit-S across occasions.

Next, the scalar invariance was examined by placing restrictions on all item intercepts to be equal over time. The scalar model provided satisfactory fit indices (CFI = 0.941, TLI = 0.931, and RMSEA = 0.050) and showed a non-significant change in CFI, TLI, and RMSEA (△CFI = −0.008, △TLI = −0.005, and △RMSEA = 0.002). Thus, the scalar invariance of the Grit-S scores also held over time.

Finally, the item uniqueness was set to be equal to test for strict invariance over time. The fit indices were adequate (CFI = 0.938, TLI = 0.933, and RMSEA = 0.049), with inappreciable differences shown in CFI, TLI, and RMSEA between the scalar and strict models (△CFI = −0.003, △TLI = 0.002, and △RMSEA = −0.001). The strict invariance of the Grit-S scores was therefore supported across time.

In sum, these results suggest that the two-factor solution of the Grit-S had LMI over the 3 months. The standardized factor loadings for the longitudinal invariance model are shown in Table 4.

**TABLE 4:** Standardized factor loadings for the longitudinal invariance model of the Grit-S.

Item	Time 1		Time 2	
	CI-1	PE-1	CI-2	PE-2
1. New ideas and projects sometimes distract me from previous ones.	0.463***		0.436***	
3. I have been obsessed with a certain idea or project for a short time but later lost interest.	0.606***		0.578***	
5. I often set a goal but later choose to pursue a different one.	0.763***		0.739***	
6. I have difficulty maintaining my focus on projects that take more than a few months to complete.	0.796***		0.775***	
2. Setbacks don’t discourage me.		0.560***		0.541***
4. I am a hard worker.		0.700***		0.682***
7. I finish whatever I begin.		0.715***		0.698***
8. I am diligent.		0.842***		0.830***

*Grit-S, the Short Grit Scale; CI-1, consistency of interest at Time 1; PE-1, perseverance of effort at Time 1; CI-2, consistency of interest at Time 2; PE-2, perseverance of effort at Time 2; ^∗∗∗^, *p* < 0.001.*

### Internal Consistency, Stability Coefficients, and Latent Factor Means Across Time

Regarding internal consistency indices, the coefficient αs for the Grit-S factor scores were acceptable (α > 0.70) at each time point in measurement. For the CI factor, the coefficient αs were 0.75 (MIC = 0.42) at Time 1 and 0.75 (MIC = 0.43) at Time 2. For the PE factor, the coefficient αs at the two measurement points were 0.80 (MIC = 0.49) at the baseline and 0.78 (MIC = 0.48) at the follow-up, respectively. Moreover, the stability coefficients (the correlations between the two time point factors) across time were computed using the strict invariance model. The resulting estimated factor correlations between Time 1 and Time 2 were 0.48 for CI and 0.66 for PE (*ps* < 0.001). Finally, the means of each latent factor at two separate time points could be made meaningfully comparison because the strict longitudinal invariance model was existed. Specifically, the latent means were not significantly different between Time 1 and Time 2 (e.g., CI mean difference = −0.013, *p* = 0.626; PE mean difference = −0.021, *p* = 0.524). Overall, these results support the stability of the Grit-S scores.

## Discussion

The purpose of the current investigation was to further explore the LMI of the Grit-S (Duckworth and Quinn, 2009), a popular instrument designed to evaluate one’s consistency of interest and PE in measuring one’s level of grit. Although some controversies with the Grit-S remain, we would like to test the longitudinal properties of the Grit-S within the two-factor solution. These findings support that the Grit-S has strict longitudinal invariance, showing equality of factor patterns, factor loadings, item intercepts and item uniqueness for all items over a 3-month interval. Moreover, the internal consistencies, stable coefficients, and latent factor means also provide the support for the stability of the Grit-S scores across time. In summary, our findings replicate and extend prior work (e.g., Duckworth and Quinn, 2009; Schmidt et al., 2017; Zhong et al., 2018) that also support the psychometric properties of Grit-S scores.

### Longitudinal Measurement Invariance of the Grit-S

Longitudinal measurement invariance assesses whether the same constructs are measured equally in different time points within a same group to ensure that growth and/or development in observed scores over time can be attributed to actual development and/or changes in the construct under investigation (Dimitrov, 2010; Millsap and Cham, 2012). Despite the fact that the psychometric properties of Grit-S scores have been supported in cross-sectional data (Duckworth and Quinn, 2009; Schmidt et al., 2017; Zhong et al., 2018), not much literature to date has addressed the longitudinal properties of the Grit-S. The present study thus examined the LMI of the Grit-S in young adults.

Similar to previous research which measured Grit-S invariance across gender and age groups (Zhong et al., 2018), the results of this current study show strict longitudinal invariance (specifically configural, metric, scalar, and strict invariance) in Grit-S scores measured across time in each of the eight items that make up the measure, suggesting that the Grit-S does indeed assess grit constructs across different moments in time. This implies that when using the Grit-S at two different time points, the mean differences in grit scores can be considered as being actual changes in an individual’s level of grit. The LMI findings also hold great significance for longitudinal research regarding the Girt-S. For instance, in longitudinal models, the input matrix becomes enormous due to numerous measurement occasions. Item parceling is often conducted to deal with this issue, whereas the use of parcels as indicators may affect MI tests at an item parcel level (Meade and Kroustalis, 2006). Therefore, achieving strict longitudinal invariance of the Grit-S at an item level in the present study supports the allowance of using item parcel sets in longitudinal models. Likewise, the Grit-S LMI is especially relevant for developmental and personality psychologists who are interested in grit. One may focus on the development and growth of one’s level of grit, while the other would be more concerned whether one’s level of grit is relatively stable or changing. Given that, until now, few studies have formally and comprehensively examined the LMI of Grit-S scores, further study on this topic is needed to ascertain the viability of the current findings in various populations (e.g., adolescents).

### Internal Consistency, Stable Coefficients, and Latent Factor Means Comparison Over Time

The internal consistency values over time also offered some meaningful information regarding the stability for Grit-S scores. Similar to cross-sectional investigations (Duckworth and Quinn, 2009; Nishikawa et al., 2015; Sarıçam et al., 2015; Li et al., 2018; Zhong et al., 2018), the coefficient αs of the Grit-S factor scores were acceptable over time, and the MIC values were adequate in the current study. Overall, our findings demonstrate that Grit-S scores have satisfactory and acceptable internal consistency indices over different periods of time.

In addition, the stability coefficients over time were computed with the LMI. More specifically, the stable coefficients that involved latent factor correlations between Time 1 and Time 2 were moderate (*r*s ranging from 0.48 to 0.66). Comparable with manifest factor correlations (Duckworth and Quinn, 2009; Li et al., 2018), the latent factor correlations also suggest that grit is somewhat stable over different measurement occasions (Duckworth et al., 2007; Duckworth and Quinn, 2009). Likewise, it is noteworthy that the test-retest reliability (particularly for consistency of interest) was not satisfactory in comparison to the rank-order consistencies found within other personality traits in young adulthood (e.g., Roberts and DelVecchio, 2000; Robins et al., 2001). In sum, our findings preliminarily support some stability (especially for PE) but also point to rank-level changes in the grit scores across the 3 months.

Finally, considering that the LMI of the Grit-S is supported, further comparisons of the latent factor means make us obtain more meaningful information. In the sample used for this study, both two grit factors (e.g., consistency of interest and PE) were not significantly different between Time 1 and Time 2. According to Duckworth et al. (2007), an important predictor of success and performance is a personality trait termed as grit, and the grit construct is defined as trait-level perseverance and a passion for long-term goals. It has been suggested that personality traits such as grit describe tendencies to act, think, and feel that are relatively stable across time (Duckworth et al., 2007; Duckworth and Quinn, 2009). Despite the fact that studies into longitudinal differences in grit are rare, our findings indicate the importance of considering both perseverance and passion for long-term goals within different contexts. Future research should examine changes in individual- and population-reported grit across time.

### Limitations and Future Directions

The findings from this study should be considered in light of its limitations. First, the participants in the present study were recruited predominantly from Southwest China, so the results may not be appropriate for other geographic regions or cultures; more research should replicate our findings in other Chinese regions. Second, we only tested the LMI of Grit-S scores over a 3-month interval; future research should test the longitudinal invariance of the Grit-S over a longer time interval. Finally, the current investigation examined longitudinal invariance of the Grit-S in young adults; future studies should test the Grit-S LMI in other populations (e.g., adolescents).

In general, the present study expands our perception of the longitudinal properties of the Grit-S measure. Moreover, we would stress that LMI is an important psychometric property of the Grit-S, particularly when it is administered in longitudinal studies looking into how grit might predict success and performance. Future work should pay further attention to this property of the Grit-S.

## Data Availability Statement

The datasets generated for this study are available on request to the corresponding authors.

## Ethics Statement

The studies involving human participants were reviewed and approved by The Human Subjects Review Committee at Guizhou Normal University. The patients/participants provided their written informed consent to participate in this study.

## Author Contributions

JL, WC, and SX contributed to the investigation, analysis of the data, and drafted the manuscript. M-CW and YG helped to perform the revision of the manuscript, and provided final approval for the manuscript.

## Conflict of Interest

The authors declare that the research was conducted in the absence of any commercial or financial relationships that could be construed as a potential conflict of interest.
